# Effect of exercise on postoperative recovery of patients with non-small cell lung cancer: a systematic review and meta-analysis

**DOI:** 10.1007/s12672-024-01079-w

**Published:** 2024-06-17

**Authors:** Mingyue Jiao, Hanping Liang, Mengge Zhang

**Affiliations:** 1 School of Teacher Education, Hezhou University, Hezhou, 542899 Guangxi China; 2 School of Tourism and Sports Health, Hezhou University, Hezhou, 542899 Guangxi China; 3 West Campus, Hezhou University, 3261 Xiaohe Avenue, Babu District, Hezhou City, Guangxi China

## Abstract

Patients with non-small cell lung cancer (NSCLC) in the postoperative recovery period often experience reduced exercise capacity and impaired lung function, which affects their overall quality of life. This paper investigated the effect of exercise interventions on exercise capacity, lung function, quality of life, and symptoms in these patients. Methods: We performed a literature search across Cochrane, Embase, PubMed, Web of Science, and EBSCO databases were comprehensively searched for randomized controlled trials (RCTs) from inception to September 2023, all English RCTs were eligible if they assessed the effects of exercise interventions on postoperative NSCLC patients. Results: Twelve articles met our inclusion criteria, evidencing that exercise interventions could significantly improve the functional capacity of NSCLC patients in postoperative recovery. Notably, Forced Expiratory Volume in 1 s (FEV1) was improved, indicating enhanced lung function. Furthermore, exercise improved the physical and mental health scores of SF-36, along with increased quadriceps strength and relieved dyspnea. However, fatigue levels were not significantly changed. Conclusions: Exercise interventions of NSCLC patients in the postoperative recovery are associated with improved functional capacity, lung function, quality of life, and quadriceps strength, as well as alleviated symptoms of dyspnea. These findings underscore the potential benefits of incorporating exercise into postoperative care for NSCLC patients. Nonetheless, further large-scale RCTs are required to solidify the evidence base on the clinical outcomes of exercise following pneumonectomy.

## Introduction

Lung cancer is the foremost cause of mortality among malignant neoplasms, with the highest incidence and prevalence [1]. According to global cancer statistics from 185 nations, lung cancer is the second most prevalent malignancy, accounting for 11.4% of all reported cases. According to the latest data (2020), lung cancer stands out as the most frequent cancer for males and the third most prevalent for females. Moreover, its mortality is significantly high, contributing to 18.0% of all cancer-related deaths [2]. Among various histological subtypes of lung cancer, non-small cell lung carcinoma (NSCLC) represents the predominant subtype with an incidence of approximately 85% to 90% [3].

Surgery is primarily to prolong the survival of lung cancer patients. Long-term survival outcomes and potential disability risks of surgery are increasingly significant for lung cancer patients [4]. Complete surgical resection remains the most effective option for stage I and II lung cancer [5]. However, systemic inflammation, impaired physical condition, inadequate nutritional status, and surgical stress contribute to postoperative functional decline, which is a crucial predictor of morbidity and mortality after pneumonectomy [6–8]. A longitudinal study conducted by Handy et al. showed a significant decline in quality of life (QOL) in 139 patients within six months after pneumonectomy [9]. Furthermore, dyspnea is strongly correlated with reduced QOL five years after surgery [10]. Additionally, dyspnea may result in decreased physical activity [11], which in turn affects skeletal muscle and cardiovascular function [12, 13], ultimately hindering the exercise capacity of lung cancer patients. Additionally, lung cancer patients may experience weight loss, loss of appetite, anemia, protein catabolism, and muscle atrophy [14, 15]. These factors all negatively impact exercise capacity. Furthermore, adjuvant treatments like chemotherapy may trigger exercise intolerance due to alterations in substrate transportation and utilization in the body [16]. Therefore, it is imperative to improve exercise capacity and QOL and alleviate associated symptoms in postoperative NSCLC patients.

Numerous studies have highlighted the importance of exercise in maintaining overall health. Specifically, both exercise training [4] and respiratory training [17] have positive effects on postoperative recovery of NSCLC patients. However, there is no consensus on the impact of exercise on postoperative outcomes in NSCLC patients [4, 18]. Granger conducted a meta-analysis in 2011 to examine the effect of exercise on NSCLC patients and unraveled that exercise capacity was improved, but QOL was not immediately improved [19]. Most studies included were case series (n = 9), with only two RCTs, potentially introducing bias and limiting the reliability and generalizability of findings. In contrast, Cavalieri (2019) conducted a meta-analysis on postoperative NSCLC patients and evinced that exercise improved both exercise capacity and QOL [20]. Nevertheless, the original studies included in Cavalheri’s meta-analysis were outdated and inadequate, casting doubt on their suitability for analyzing pooled results [20]. Specifically, some outcome measures reported final values after exercise rather than within-group differences (changes from baseline to post-intervention), which reduced the credibility of the combined results and increased the likelihood of bias. Moreover, this analysis did not distinguish between different types of exercise, thereby constraining our comprehension of how specific exercise affect postoperative recovery in NSCLC patients and hindering the development of personalized exercise programs for clinical application. Given the large number of recent research on the effects of exercise on postoperative NSCLC patients, a thorough review of the latest studies is crucial to elucidate the benefits of exercise for this population.

This meta-analysis of current data was to elucidate the impact of exercise on postoperative NSCLC patients. Different from recent studies, this innovative research incorporated the most up-to-date studies from the past five years and analyzed baseline and post-intervention changes. Additionally, subgroup analyses were implemented to assess exercise capacity and QOL. Given that lung function, exercise capacity, QOL, and cancer-related symptoms are crucial influencing factors for postoperative recovery in NSCLC patients [21] and that data on other factors are limited, this meta-analysis was to comprehensively summarize the effects of exercise on lung function, exercise capacity, QOL, and cancer-related symptoms in postoperative NSCLC patients.

## Materials and methods

This meta-analysis followed the PRISMA guidelines [22], and the systematic review has also been registered on PROSPERO (CRD42023488002).

### Data sources and searches

Relevant studies were comprehensively searched on PubMed, Cochrane, Embase, Web of Science, and EBSCO databases. The search strategy utilized MeSH terms in PubMed/Cochrane and Emtree terms in Embase.

Appendix S2 gives details of the search strategy for each database.

The search strategy was based on the PICOS: (P) Population: NSCLC patients; (I) Intervention: exercise; (C) Controls: usual care, or no exercise training, or only general exercise instructions; (O) Outcome: postoperative recovery: pulmonary function, exercise capacity, QOL, muscle strength, dyspnea, and fatigue; (5) Study type: RCT. In addition, we searched the reference lists of selected articles to identify any relevant studies that might have been missed by the electronic search. All English RCTs published from database inception to September 2023 were included.

### Study selection

The literature was screened by two independent researchers based on predefined inclusion and exclusion criteria using a double-blind approach. Initially, articles were selected by reviewing the title and abstract, followed by full-text reading to determine the inclusion. Any disagreement was addressed by a third author who made the final decision. Eligible studies met the following criteria: (1) RCTs, (2) participants aged ≥ 18 years undergoing pneumonectomy, (3) control groups receiving usual or standard care without exercise training or with only general exercise instruction, (4) interventions involving aerobic training, resistance training, strength training, or respiratory exercises combined with other exercises, (5) comparison of experimental groups receiving structured exercise training for at least four weeks, (6) assessment of changes in factors related to postoperative recovery before and after exercise, and (7) reporting of at least one outcome measure related to pulmonary function, exercise capacity, or QOL. Studies were excluded for duplicate publications, literature reviews, letters to the editor, conference abstracts, assessing acute effects of single bouts of exercise, animal studies, and lacking primary data or where attempts to contact authors proved unsuccessful.

### Data extraction and quality assessment

All data were extracted and compiled independently by two researchers to ensure consistency. A third author was asked for help to make the final decision in case of disagreement. If relevant data were missing, we contacted the authors to obtain the original information. The extracted information encompassed lead author details, country of affiliation, year of publication, TNM cancer stage; baseline characteristics: age, sample size, and type of surgery (open thoracic or minimally invasive) in both experimental and control groups; exercise characteristics: type, frequency, duration, and intensity of intervention; and reported outcomes. The risk of bias was appraised using the Cochrane Bias risk tool [23] in seven aspects: (1) randomization generation, (2) concealment of allocation, (3) blinding of participants and personnel, (4) blinding of outcome assessment, (5) incomplete outcome data, (6) selective reporting bias, and (7) other bias.

### Data synthesis and analysis

Odds ratios (ORs) with 95% confidence intervals (CI) were utilized as pooled estimates for dichotomous outcomes. Conversely, mean differences (MDs) accompanied by a 95% CI were computed for continuous outcomes. Due to similar units of measurement and statistical methods of most data, MDs and their 95% CI were adopted. However, for a small proportion of data with different units of measurement, standardized mean differences (SMDs) and 95% CI were calculated. Changes in the intervention group compared to the control group were also summarized to estimate the effect of each outcome. Heterogeneity across studies was judged using the I^2^ statistic and Cochran’s Q test. Significant heterogeneity was considered present when I^2^ > 50% or when the p-value from the Q test ≤ 0.10. Random-effects models were employed if significant heterogeneity was discovered; otherwise, fixed-effects models were used. Subgroup analyses were conducted to ascertain sources of significant heterogeneity while simultaneously examining the effects of different exercise modalities. Publication bias was discerned by funnel plots and Egger’s and Begg’s methods. Sensitivity analyses tested the robustness of pooled results by excluding each RCT. Quantitative syntheses of data were done using Review Manager software 5.3 or Stata software 17.

## Results

### Literature selection

**Fig. 1:**
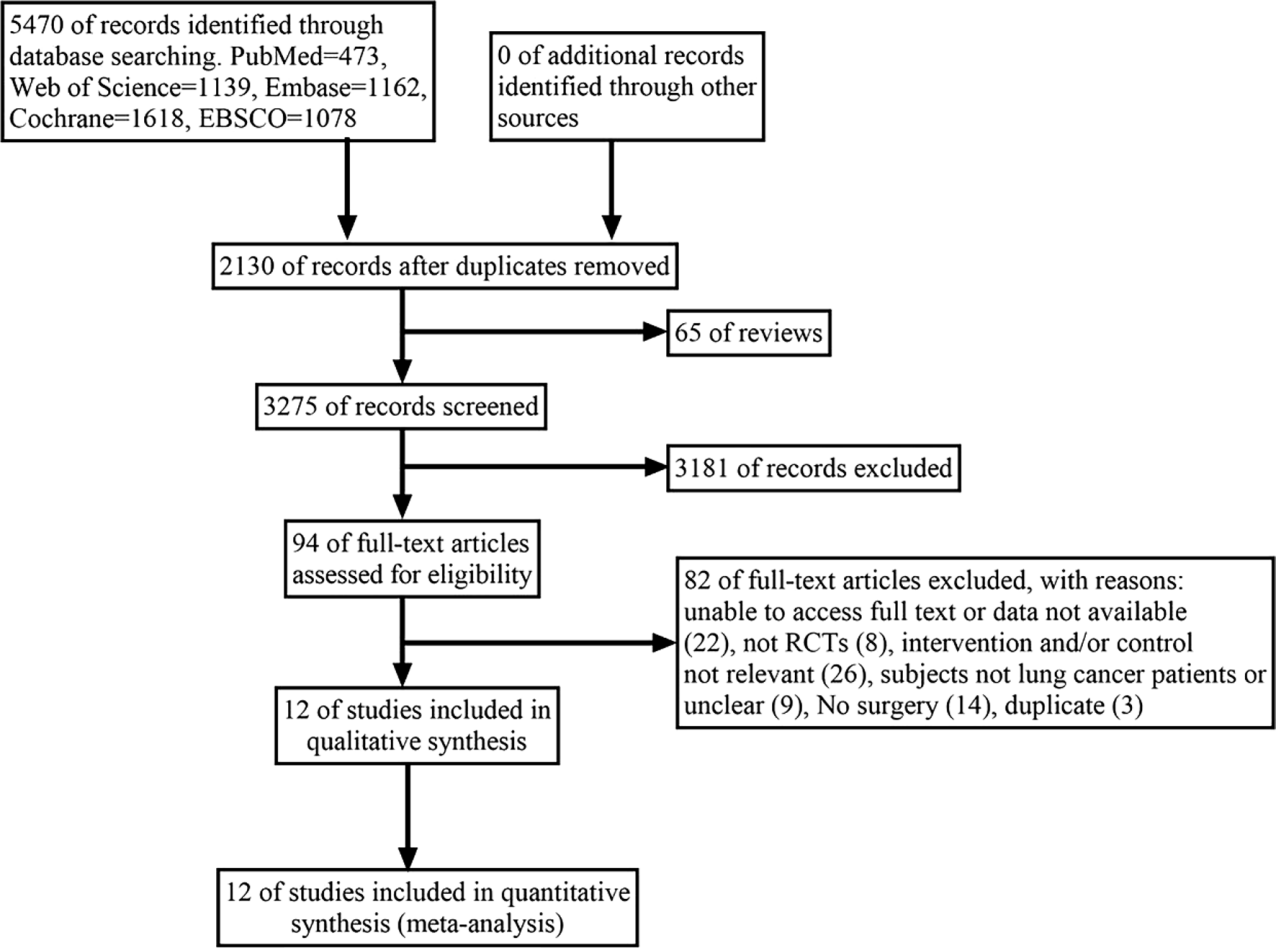
Flow chart of the study selection process

The flowchart of selection process is presented in Fig. 1. 5470 potentially eligible articles were retrieved from which duplicates and reviews were removed, resulting in 3275 articles for screening. After evaluating titles and abstracts, 3181 articles were excluded, followed by the removal of 82 articles after reading their full texts. Ultimately, only 12 eligible articles were enrolled.

### Description of the included RCTs

#### Participants

The characteristics of the included RCTs are summarized in Table 1. 413 cases in the experimental group and 407 cases in the control group were included. Ten RCTs included only postoperative NSCLC patients. Two RCTs included postoperative patients with NSCLC and small cell lung cancer (SCLC). Ten RCTs reported the surgical approach used, video-assisted thoracoscopic surgery (VATS) or open thoracic surgery. The mean age of the study participants ranged from 56 to 68 years, with the majority being middle-aged or older.

**Table 1:** Characteristics of included RCTs

Study	Country	Age, mean, years (I/C)	Sample size	Operation type	TNM cancer stage	Intervention	Initial timing of intervention	Intensity	Training Time (min/day)	Sessions (n/week)	Duration (weeks)	Control	Outcomes
Arbane 2011 [4]	UK	65.4/62.6	IG: 26, CG: 25	Open: 51	NSCLC	AE, RE	First day after surgery	walking, marching on the spot, and recumbent bike 60–80% MHR (5 min)	> 5	7	12	Usual care	③⑥⑦
Granger 2013 [24]	Australia	57.0/72.4	IG: 7, CG: 8	–	I, II, III, IV NSCLC	AE, RE	First day after surgery	AE: walking, cycling (5–15 min) RE: knee lifts, squats, step-ups, sit to stands, and heel raises	30	Before discharge: 14, after discharger: 2	8	Respiratory physiotherapy	③④⑥
Stigt 2013 [18]	Netherlands	63.6/63.2	IG: 23, CG: 26	Open: 47, VATS: 2	NSCLC	AE, RE	Four weeks after surgery	Cycling 60–80% peak load	60	2	12	Usual care	①③④
Arbane 2014 [25]	UK	67/68	IG: 64, CG: 67	Open: 90, VATS: 38	I, II, III, IV NSCLC	AE, RE	First day after surgery	unloaded pedalling 60–90% MHR	30	7	4	Usual care	⑥⑦
Brocki 2014 [17]	Denmark	64/65	IG: 41, CG: 37	Open: 60, VATS: 18	I, II, III NSCLC	AE, RE	Three weeks after surgery	warming up: 15 minAE: stationary bike (20 min) RE: 2 ∗ 10 Arms, Trunk, Legs (15 min), Cooling down: 5 min	60	1	10	Unsupervised exercise training	①③④
Edvardsen 2015 [26]	Norway	64.4/65.9	IG: 30,CG: 31	–	I–IV NSCLC	HIIT, RE	Five-seven weeks after surgery	HIIT: Walking uphill on a treadmill 80%–95% HRmax RE: 3*6–12 RM upper and lower limb, back strength	60	3	20	Standard postoperative care	①⑤
Salhi 2015 [27]	Belgium	65/65	IG: 16,CG: 10	–	I, II, III NSCLC, SCLC	AE, RE	First day after surgery	AE: bicycle, treadmill 70% of the respective maximal workload (Wmax) and speed(20 min) RE: (3 ∗ 8) 50% 1-repetition-maximum	30	3	12	Unsupervised exercise training	③⑤⑥⑧⑨
Cavalheri 2017 [28]	Australia	66/68	IG: 9, CG: 8	Open: 8, VATS: 9	I, II, IIIa NSCLC	HIIT, RE	4 weeks after surgery	HIIT: 20 min walking 70/80% 6MWD speed or 10 min cycling 80% WRpeak RE: 3 ∗ 10 upper limb training	60	3	8	Usual care	③④⑤⑥⑧
Messaggi-sartor 2019 [29]	Spain	64.2/64.8	IG: 16, CG: 21	Open: 34,VATS: 3	I or II NSCLC	AE, HIRMT, RE	First day after surgery	Warm-up: 5 min AE: cycling 60% Wpeak (30 min) HIIT: IEMT (5 ∗ 10 breathing) 50%PImax/PEmax (15 min) RE: bicep curl, chest, and shoulder press Cool down: 5 min	60	3	8	Usual care	⑤⑥
Quist 2018 [30]	Denmark		IG: 110, CG: 101	Open: 39, VATS: 172	I-IIIaNSCLC	AE, RE	Two weeks after surgery	Early strength exercise and cardiovascularexercise (twice a week (60 min/sessions))	60	2	12	Usual care	③④⑤⑥
Liu 2021 [31]	China	64.2/66.3	IG: 26, CG: 28	VATS: 54	Ia, Ib, IIa, IIb, IIIa, NSCLC, SCLC	inspiratory muscle training, AE	beginning on the day of chest tube removal	IMT: 2*30 moderate intense AE: 50–70% of the patient’s exercise capacity (60 min)	60	Twice daily	6	Standard care	③
Zou 2022 [32]	China	60.09/56.84	IG: 45, CG: 45	VATS: 90	NSCLC	APVPT BE, AE	First day after surgery	APVPT: 2*20 positive expiratory pressure breathing exercises(8 min) BE: 3 ∗ 15 Upper and lower limb training (15 min) AE: cycling(20 min), Dancing(60 min)	60	3 times a day	13	Usual care	①③

NSCLC, non-small cell lung cancer; SCLC, small cell lung cancer; ①FEV1, forced expiratory volume in 1 s; ②FEV, forced expiratory volume; ③6MWD, six-minute walk distance; ④SF-36, the 36-Item Short Form Health Survey; ⑤VO_2_peak, peak oxygen uptake; ⑥EORTC QLQ-C30, The European Organization for Research and Treatment of Cancer; ⑦quadriceps; ⑧fatigue; ⑨dyspnoea; VATS, video-assisted thoracoscopic surgery; AE, aerobic exercise; RE, resistance exercise; HIIT, high-intensity interval training; IEMT, inspiratory and expiratory muscle training; APVPT, acapella positive vibration pressure training; MHR, maximal heart rate; WRpeak, peak work rate; Wpeak, peak workload

#### Interventions

A concise overview of exercise programs is presented in Table 1. Twelve studies implemented exercise interventions during the postoperative period, encompassing six articles using aerobic and resistance exercises, two articles using HIIT with resistance exercise, one article combining aerobic, HIIT, and resistance exercises, one article combining resistance and cardiovascular exercises, one article combining aerobic, resistance, and respiratory exercises, and one article solely on aerobic and respiratory exercises. The primary forms of aerobic exercise were cycling, walking, and treadmill activities. The main form of resistance training was the range of motion exercises for the trunk and limbs. Chest breathing techniques, abdominal breathing methods, or ventilator assistance, were primary forms of respiratory exercise. All interventions lasted at least four weeks. During this period, the frequency of each exercise intervention varied. The control group in each study received either standard care no, specific exercise training, or only general instructions regarding physical activity.

#### Outcome

The outcome measures are presented in Table 1. These studies primarily focused on pulmonary function [e.g., forced expiratory volume (FEV), forced expiratory volume in 1 s (FEV1)], exercise capacity [e.g., six-minute walk distance (6MWD), peak oxygen uptake (VO_2_peak)], QOL [the 36-item short form health survey (SF-36), the European organization for research and treatment of cancer (EORTC QLQ-C30)], muscle strength (e.g., quadriceps, grip strength), dyspnea, fatigue, postoperative complications, and length of hospitalization. Two studies examined the impact of exercise on forced vital capacity (FVC) and various aspects of functional status, symptoms, role functioning, cognitive functioning, social functioning, grip strength, handgrip strength, anxiety levels, depression levels, and oxygen saturation (SPO_2_) in postoperative lung cancer patients. Due to limited data available for FVC, postoperative complications, functional status, symptoms, role functioning, cognitive functioning, social functioning, anxiety levels, depression levels, and SPO_2_ from the EORTC QLQ-C30 questionnaire were not included in this analysis. Additionally, other outcome indicators such as the ratio of spirometry to airflow capacity (FEV1/FVC), diffusion function, and St George’s respiratory questionnaire (SGRQ) were studied but could not be analyzed due to insufficient data.

### Methodological quality assessment

**Fig. 2:**
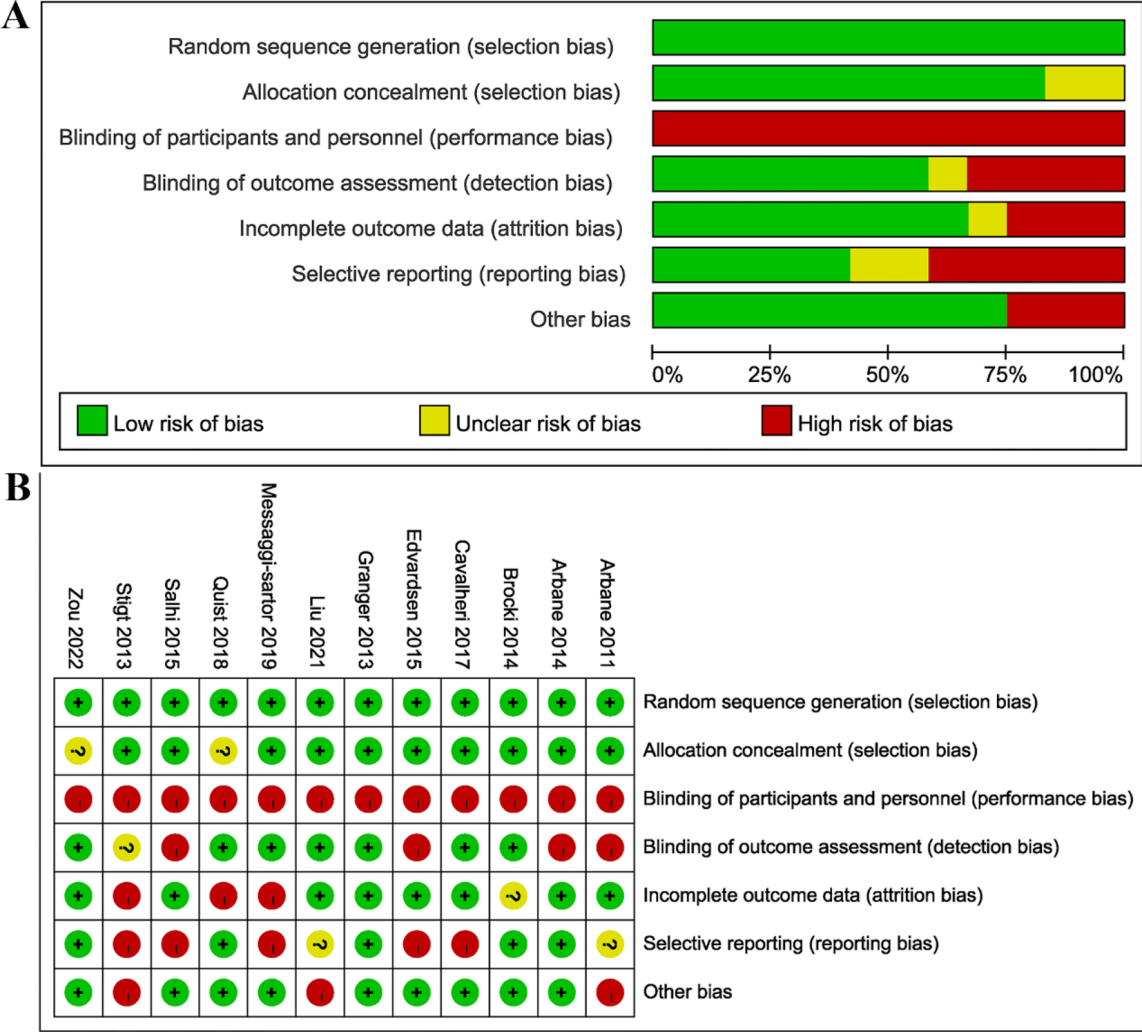
Cochrane risk bias evaluation chart. **A** Risk of bias graph **B** Risk of bias summary

The results of Cochrane risk evaluation are presented in Fig. 2. Among the enrolled articles, twelve explicitly reported randomization methods and ten had concealed the allocation of groups. Given that all studies involved human subjects, blinding of participants and personnel was a challenge. However, informed consent forms were signed by participants, and exercise interventions were supervised by researchers. Consequently, all articles were deemed a high risk of bias. Seven articles described blinding for outcome analysis, but three were rated as high risk due to a significant loss of personnel during the intervention. Some papers clearly outlined how missing data were handled and the methods used, while five papers selectively reported outcomes. When reviewing the trial registers, it was found that not all prespecified results were reported in the published papers. In addition, some outcomes appeared in the papers but not in the registers, leading to three articles being rated as high risk due to baseline differences.

### Synthesis of the results

#### Analysis of pulmonary function

**Fig. 3:**
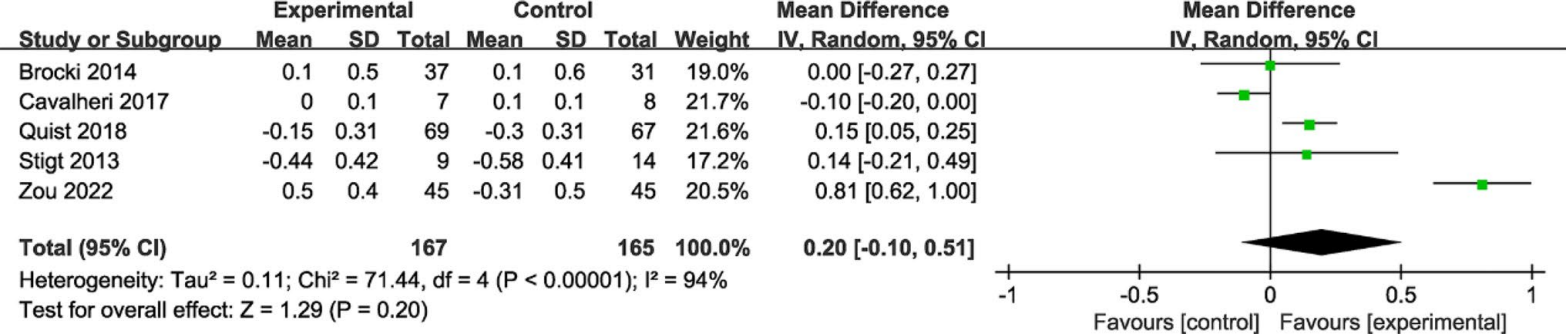
Forest plot of post-intervention FEV1 value. SD: standard deviation; IV: inverse variance; CI: confidence interval

**Fig. 4:**
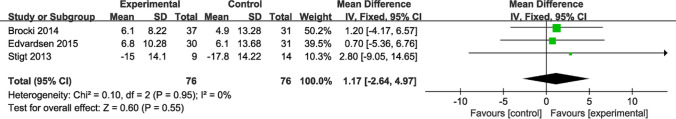
Forest plot of post-intervention FEV value. SD: standard deviation; IV: inverse variance; CI: confidence interval

Lung function was measured in six studies, with FEV1 and FEV analyzed. Among the five studies investigating FEV1, no great difference was noted between the intervention and control groups (MD = 0.20; 95% CI [− 0.10, 0.51]; *P* = 0.20; *I*^*2*^ = 94%) (Fig. 3). In three studies on FEV, there was also no marked difference (MD = 1.17; 95% CI [− 2.64, 4.97]; *P* = 0.55; *I*^*2*^ = 0%) (Fig. 4).

#### Analysis of 6MWD

**Fig. 5:**
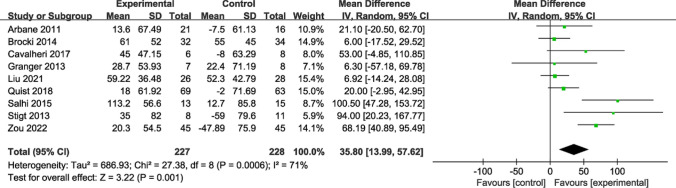
Forest plot of post-intervention 6MWD value. SD: standard deviation; IV: inverse variance; CI: confidence interval

The effect of exercise on 6MWD is summarized in Fig. 5. Nine studies with 6MWD as a measure of exercise capacity were included, including 455 participants. Due to substantial heterogeneity (*I*^*2*^ = 71%), a random-effects model was employed to estimate combined effects, and the analysis demonstrated substantial differences between the intervention and control groups (MD = 35.80; 95% CI [13.99, 57.62]; *P* = 0.001).

#### Analysis of VO_2_peak

**Fig. 6:**
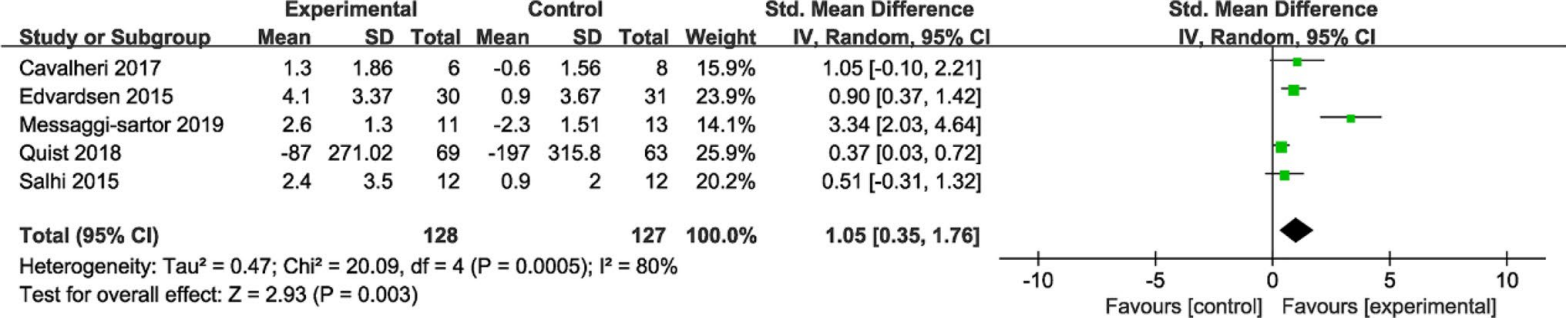
Forest plot of postintervention VO_2_peak value SD: standard deviation; IV: inverse variance; CI: confidence interval

Five studies reported VO_2_peak as an indicator of exercise capacity, with 255 participants providing data. Four of these studies utilized the statistical unit of mL/kg/min to measure VO_2_peak, while the remaining study used ml O_2_/min. Considering the different units, we employed SMD for data comparison and analysis. Due to substantial heterogeneity (*I*^*2*^ = 80%), random-effects models were applied for combined effects estimation. The results noted a significant disparity between the intervention and control groups (SMD = 1.05; 95% CI [0.35, 1.76]; *P* = 0.003) (Fig. 6). Sensitivity analyses were conducted to identify potential sources of heterogeneity.

#### Analysis of QOL

To comprehensively investigate the impact of postoperative exercise on QOL in patients undergoing pneumonectomy, we conducted a statistical analysis using SF-36 and EORTC QLQ-C30.

Six studies assessed QOL using the SF-36 questionnaire. The pooled results demonstrated a significantly improved physical domain score of SF-36 after postoperative exercise (MD = 3.10, 95% CI [1.28, 4.93]; *P* = 0.0008; *I*^*2*^ = 29%) (Appendix S3-1). However, substantial heterogeneity was noticed (*I*^*2*^ = 89%). Therefore, a random-effects model was employed for analysis (MD = 4.76, 95% CI [− 1.78, 11.30]; *P* = 0.15; *I*^*2*^ = 89%). Notably, no prominent differences were found between the two groups in the psychological domains of SF-36 (Appendix S3-2).

Postoperative exercise training was significantly correlated with improved physical function (MD = 7.91, 95% CI [0.84, 14.98]; *P* = 0.03; *I*^*2*^ = 76%) and role physical (MD = 7.73, 95% CI [2.91, 12.55]; *P* = 0.002; *I*^*2*^ = 37%) (Appendix S3-3).

Postoperative motor rehabilitation was significantly correlated with social functioning (MD = 6.80, 95% CI [3.63, 9.98]; *P* < 0.0001; *I*^*2*^ = 0%) and psychological well-being (MD = 9.47, 95% CI [7.54, 11.41]; *P* < 0.00001;* I*^*2*^ = 18%) within the four psychological domains (Appendix S3-4).

Six studies reported disease-specific health-related quality of life (HRQoL) using the EORTC QLQ-C30, while five studies reported global health component scores on the same questionnaire. No noticeable differences were found in global health component scores (MD = 1.39, 95% CI [− 2.53, 5.32]; *P* = 0.49; *I*^*2*^ = 0%). However, significant differences were observed in body composition across four studies (MD = 4.88, 95% CI [2.05, 7.72]; *P* = 0.0007; *I*^*2*^ = 0%). In contrast, no significant differences were found between in three studies investigating affective functioning (MD = 11.47, 95% CI [− 4.58, 27.53]; *P* = 0.16; *I*^*2*^ = 77%) (Appendix S3-5).

#### Analysis of quadriceps

**Fig. 7:**
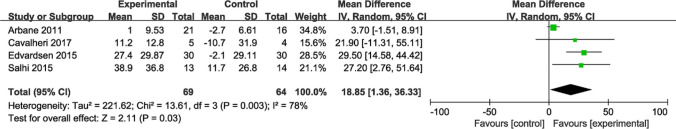
Forest plot of post-intervention quadriceps value. SD: standard deviation; IV: inverse variance; CI: confidence interval

Four studies reported the intervention's impact on quadriceps muscle, with 133 participants providing data. Substantial evidence indicated high heterogeneity (*I*^*2*^ = 78%). Consequently, a random-effects model was employed for analysis (MD = 18.85; 95% CI [1.36, 36.33]; *P* = 0.03) (Fig. 7). The findings demonstrated visible differences in quadriceps muscle, and sensitivity analysis was conducted to identify the source of heterogeneity.

#### Analysis of dyspnea

**Fig. 8:**
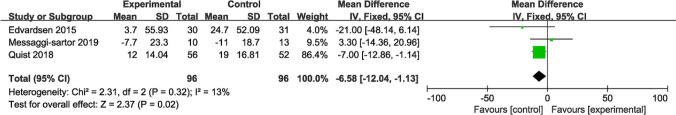
Forest plot of post-intervention dyspnea value. SD: standard deviation; IV: inverse variance; CI: confidence interval

Three studies examined the impact of interventions on dyspnea, involving 192 participants. A remarkable difference was noted between the intervention and control groups (MD = − 6.58, 95% CI [− 12.04, 1.13]; *P* = 0.02) (Fig. 8). The low heterogeneity across these studies (*I*^*2*^ = 13%, P for heterogeneity = 0.32) suggests that exercise interventions may effectively alleviate dyspnea in postoperative NSCLC patients.

#### Analysis of fatigue

**Fig. 9:**
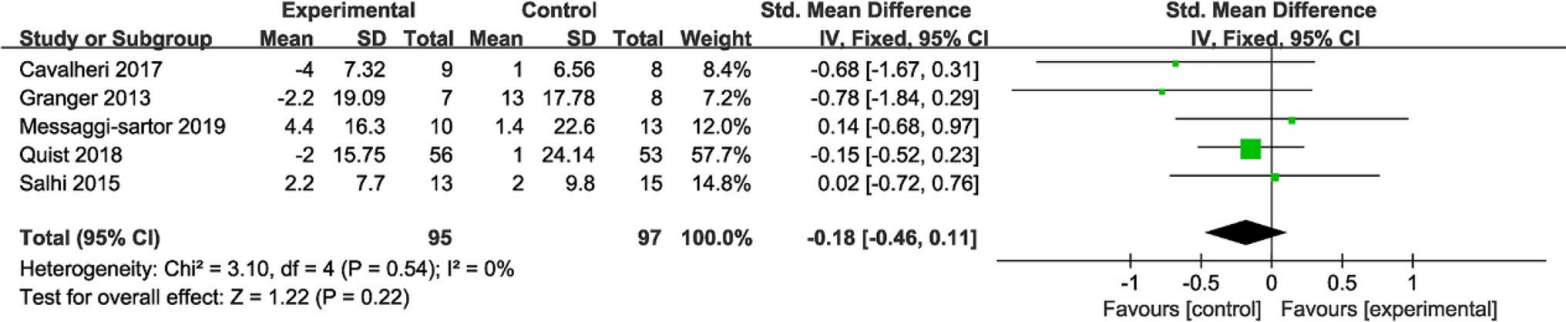
Forest plot of post-intervention fatigue value SD: standard deviation; IV: inverse variance; CI: confidence interval

Five studies with participants reported the impact of the intervention on fatigue. Among these, three studies utilized the fatigue component score of the EORTC QLQ-C30, while the remaining two employed functional assessment of chronic illness therapy—fatigue (FACIT-Fatigue) for measuring fatigue levels. Higher scores on the EORTC QLQ-C30 indicate more severe fatigue, whereas lower scores on FACIT-Fatigue suggest greater fatigue severity. During data sorting and collection, we reversed the direction of the average change in FACIT-Fatigue scores (i.e., negative values were treated as positive). Due to different assessment methods, number of questions, and score ranges, SMD was calculated to analyze the data. The results obtained through fixed-effects model analysis unveiled no significant difference in fatigue (SMD = − 0.18; 95% CI [− 0.46, 0.11]; *P* = 0.22; *I*^*2*^ = 0%) (Fig. 9).

### Subgroup analysis

#### Subgroup analysis of FEV1

**Fig. 10:**
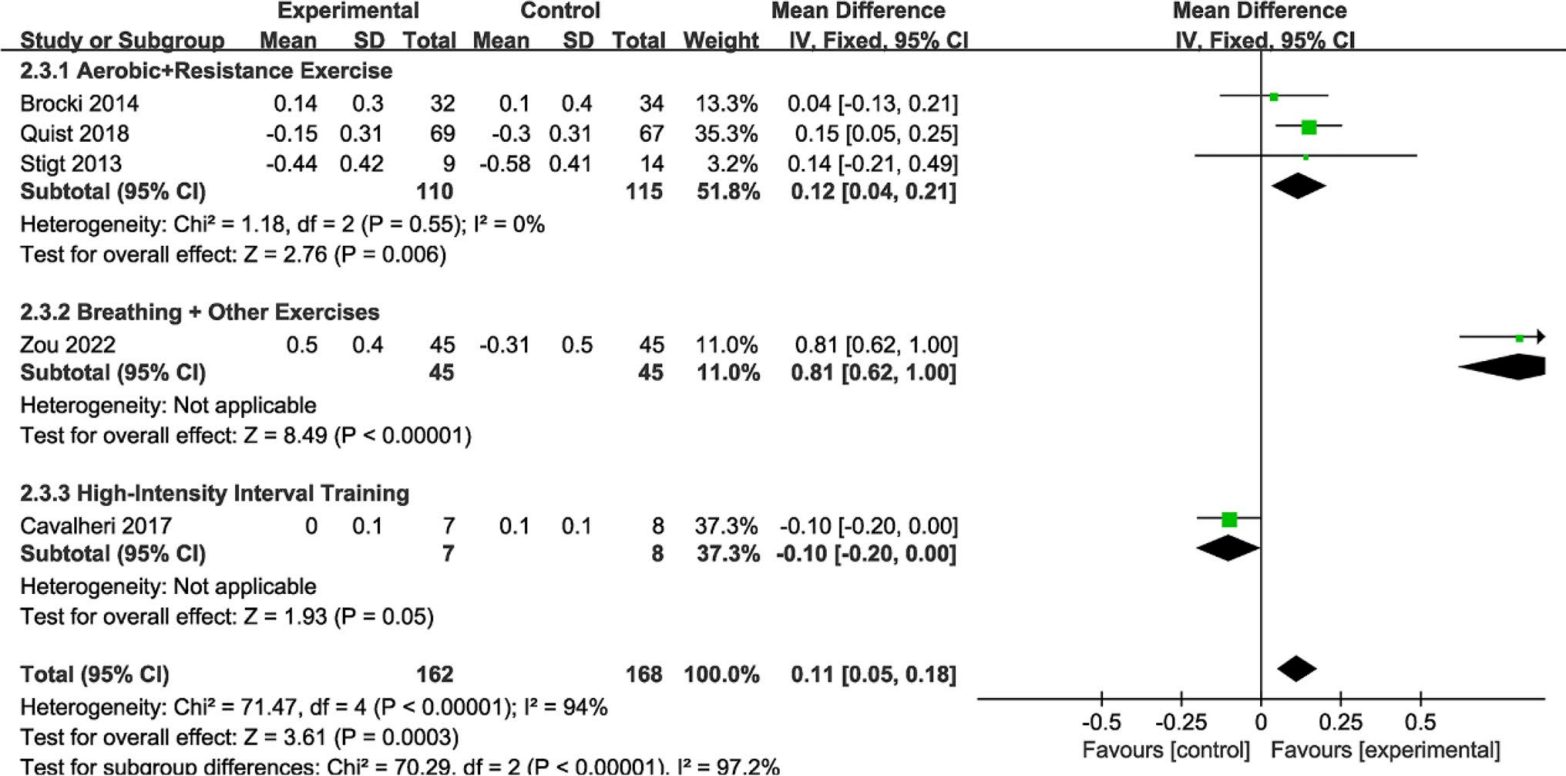
Forest plot of post-intervention FEV1 value. SD: standard deviation; IV: inverse variance; CI: confidence interval

Regarding the impact on postoperative NSCLC patients, our previous analysis of the overall sample did not disclose any significant findings (p > 0.05) in FEV1 for lung function. However, subgroup analyses (Fig. 10) unveiled some intriguing results. Three studies (n = 225 patients) reported aerobic and resistance exercise, which demonstrated notable differences between the intervention and control groups (MD = 0.12; 95% CI [0.04, 0.21]; *P* = 0.006; *I*^*2*^ = 0%). One study (n = 90 patients) provided data on FEV1 for breathing exercises combined with other forms of exercise, showing marked differences (MD = 0.81; 95% CI [0.62, 1.00]; *P* < 0.00001). Additionally, one study (n = 90 patients) presented FEV1 measurements for HIIT, revealing significant differences (MD = − 0.10; 95% CI [− 0.20, 0.00]; *P* = 0.05).

#### Subgroup analysis of 6MWD

**Fig. 11:**
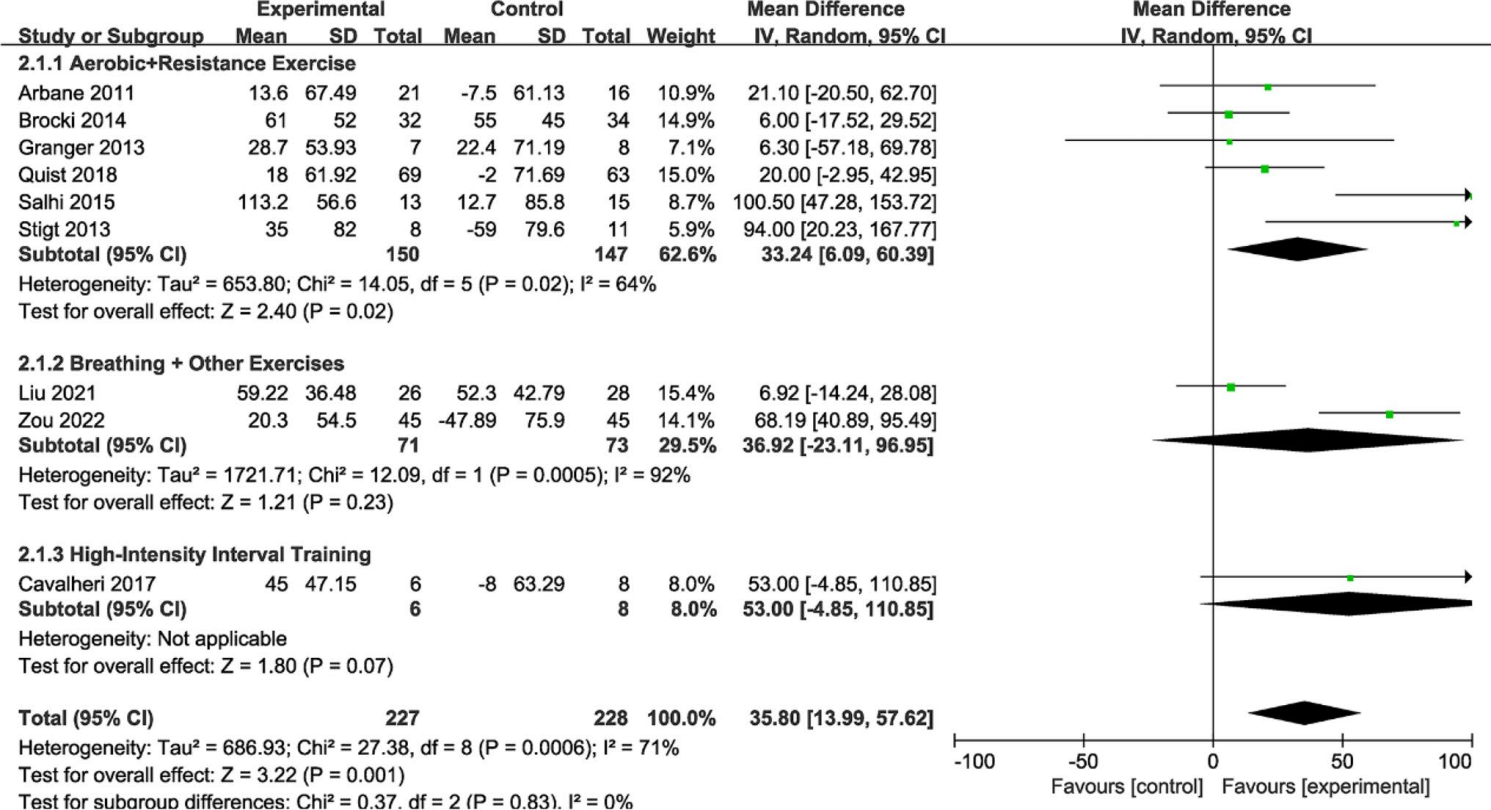
Forest plot of post-intervention 6MWD value. SD: standard deviation; IV: inverse variance; CI: confidence interval

Figure 11 presents a summary of the impact of exercise on 6MWD. A random-effects model was employed for estimating combined effects, considering data from nine RCTs involving 455 participants. Overall, exercise demonstrated a noticeable increase in 6MWD levels (MD = 35.80; 95% CI [13.99, 57.62]; *P* = 0.004;* I*^*2*^ = 73%). Subgroup analyses yielded varying results. Six studies (n = 297 patients) reporting aerobic and resistance exercises manifested significant differences (MD = 33.24; 95% CI [6.09, 60.39]; *P* = 0.02;* I*^*2*^ = 64%). However, two studies (n = 144 patients) providing data on breathing and other exercises did not show any notable differences (MD = 36.92; 95% CI [− 23.11, 96.95]; *P* = 0.23; *I*^*2*^ = 92%). Additionally, one study (n = 14 patients) examining HIIT exhibited no marked difference (MD = 53.00; 95% CI [− 4.85, 110.85]; *P* = 0.07).

#### Subgroup analysis of physical domain

**Fig. 12:**
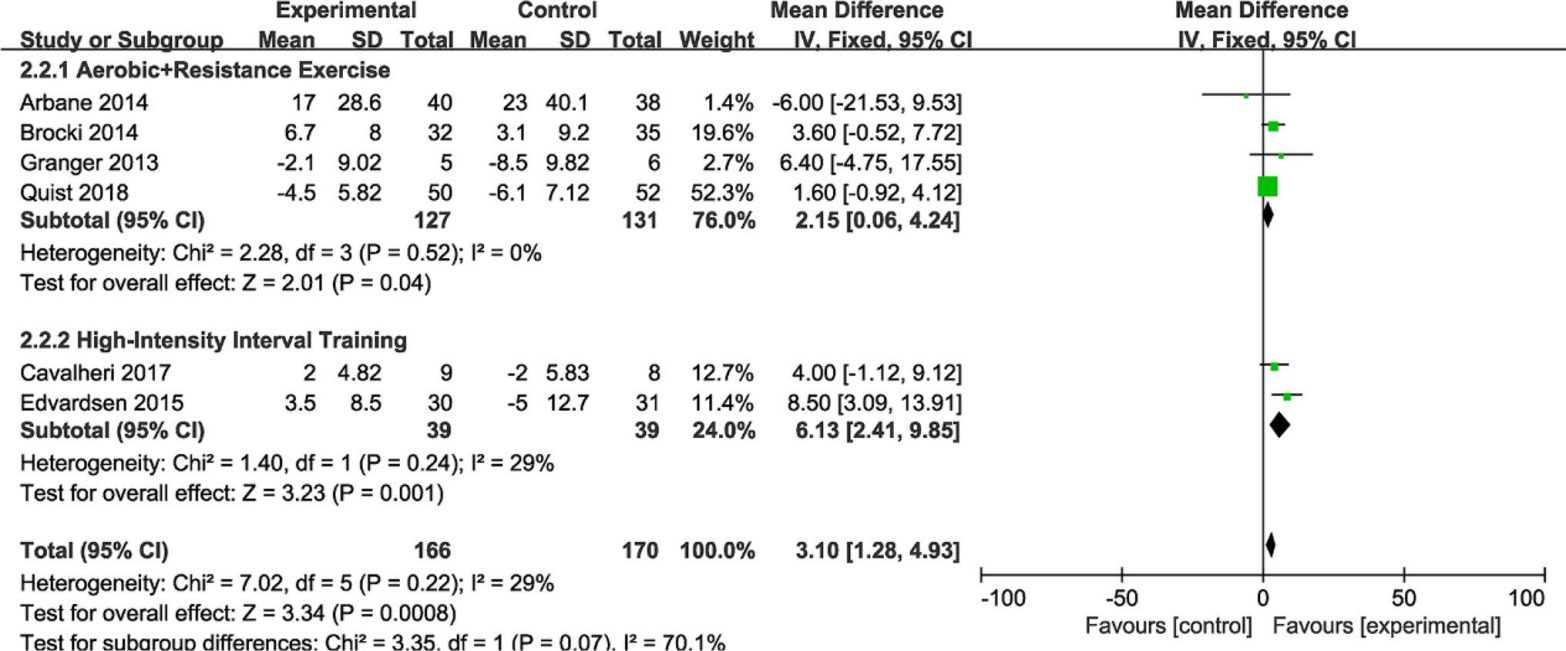
Forest plot of post-intervention physical domain value. SD: standard deviation; IV: inverse variance; CI: confidence interval

Figure 12 presents a summary of the impact of exercise on physical aspects of QOL, with random-effects models utilized for combined effects estimation. Six RCTs with 336 participants assessed the SF-36 physical domain. Overall, exercise visibly improved bodily domains (MD = 3.10; 95% CI [1.28, 4.93]; *P* = 0.00008; *I*^*2*^ = 29%). Subgroup analyses yielded consistent results. Four studies (n = 258 patients) reported physical domains combining aerobic and resistance exercise, and analyses demonstrated prominent differences (MD = 2.15; 95% CI [0.06, 4.24]; *P* = 0.04; *I*^*2*^ = 0%). Two studies (n = 78 patients) reported the physical domains of HIIT, and analysis proved substantial differences (MD = 6.13; 95% CI [2.41, 9.85]; *P* = 0.001; *I*^*2*^ = 29%).

#### Subgroup analysis of mental domain

**Fig. 13:**
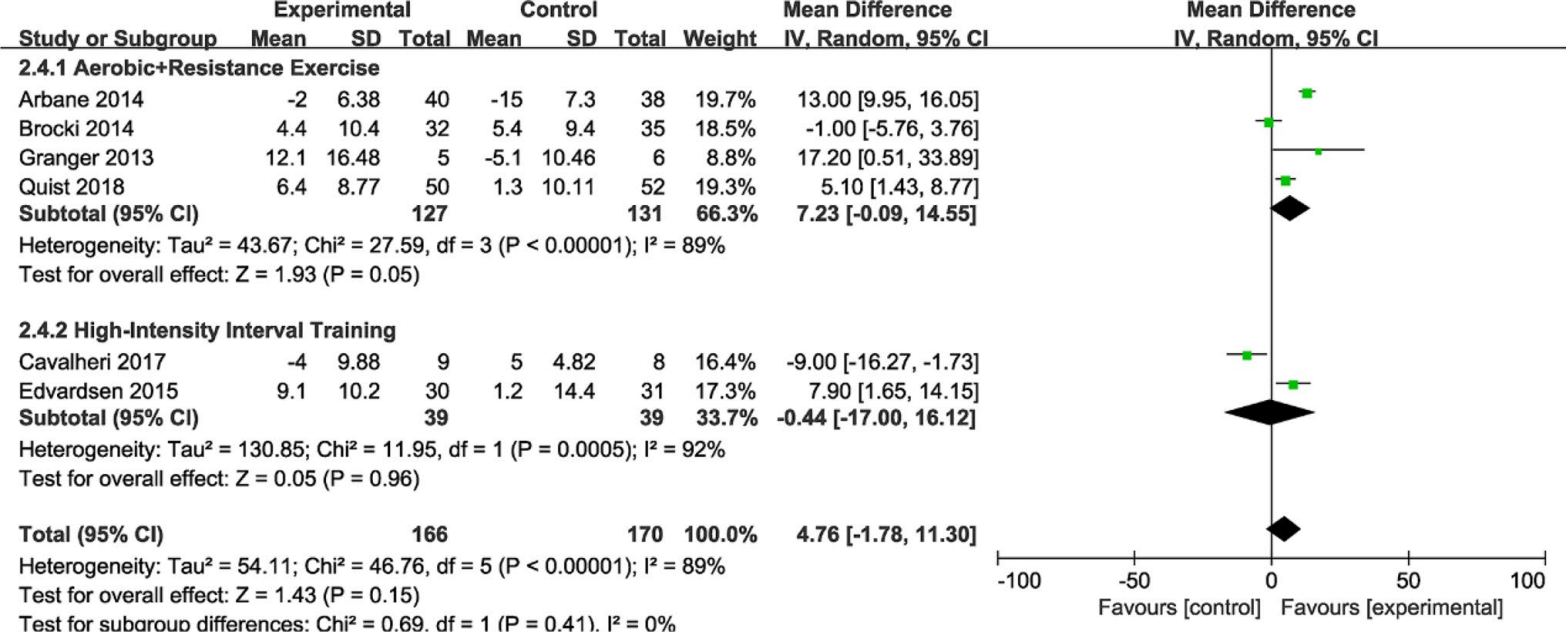
Forest plot of post-intervention mental domain value. SD: standard deviation; IV: inverse variance; CI: confidence interval

Previous results from the overall sample analysis implied no statistical significance (*P* > 0.05) for the mental domain outcome metrics of the SF-36 questionnaire. However, noteworthy findings emerged after subgroup analyses on exercise interventions (Fig. 13). Four articles (n = 258 patients) reported notable differences in the mental domain outcomes for combined aerobic and resistance exercise (MD = 7.23; 95% CI [− 0.09, 14.55]; *P* = 0.05; *I*^*2*^ = 89%). Two articles (n = 78 patients) reported no notable difference in the mental domain outcomes for HIIT (MD = − 0.44; 95% CI [− 17.00, 16.12]; *P* = 0.96; *I*^*2*^ = 92%). To comprehensively explore the impact of postoperative exercise on QOL, we conducted a statistical analysis using SF-36 and EORTC QLQ-C30.

### Sensitivity analysis

To evaluate the stability of the meta-analysis, a sensitivity analysis was conducted. After excluding the study by Messaggi-sartor 2019 [29], heterogeneity for VO_2_peak greatly decreased (*I*^*2*^ = 12%), indicating that this study contributed to the observed heterogeneity. However, there was no significant change in the adjusted combined estimate (SMD = 0.55; 95% CI [0.29, 0.82]; *P* < 0.0001). Similarly, the removal of Arbane's study led to a significant reduction in heterogeneity for SF-36 physical functional values (*I*^*2*^ = 24%) [25], but it did not result in any substantial changes in the adjusted combined estimates (MD = 4.84; 95% CI [0.02, 9.67]; *P* = 0.05). The elimination of Arbane's study [4] substantially reduced heterogeneity for quadriceps data (*I*^*2*^ = 0%), suggesting it was a major source of heterogeneity; however, there were no evident alterations in the adjusted combined estimates (MD = 27.98; 95% CI [16.09, 39.87]; *P* < 0.00001). These findings indicated that the results obtained from this meta-analysis were relatively robust.

Sensitivity analyses were done for FEV1, 6MWD, physical domain, and psychological domain. Upon exclusion of each study, the sensitivity analyses demonstrated robustness and stability in the overall findings (Appendix S4).

### Evolution of publication bias

The funnel plot showed no significant evidence of asymmetric distribution for FEV1, 6MWD, physical domain, and psychological domain. Begg's and Egger's tests indicated no significant publication bias in FEV1 (Pr >|z|= 0.624 > 0.05) (t = 1.11, *P* = 0.349), 6MWD (Pr >|z|= 0.404 > 0.05) (t = 0.39, *P* = 0.706), physical domain (Pr >|z|= 0.188 > 0.05) (t = -0.30, *P* = 0.782), and psychological domain (Pr >|z|= 0.573 > 0.05) (t = 0.74, *P* = 0.502) (Appendix S5).

## Discussion

This systematic review presents a comprehensive synthesis of recent RCTs, employing meta-analysis to examine the impact of exercise on postoperative recovery in NSCLC patients. The findings substantiate that exercise interventions offer numerous advantages for postoperative NSCLC patients, including improved FEV1, enhanced exercise capacity, better QOL across specific domains, improved quadriceps function, and alleviated dyspnea. However, no significant effects were observed on fatigue.

Many systematic reviews and meta-analyses have shown that exercise interventions can significantly improve cardiopulmonary function and reduce the risk of respiratory diseases such as upper respiratory tract infections and pneumonia [33]. However, these recent meta-analyses presented somewhat negative results when assessing the effects of exercise on the psychological domains of pulmonary function and QOL in NSCLC patients after surgery [34, 35]. However, through the inclusion of new literature and in-depth subgroup analysis, we observed encouraging findings. The combination of aerobic and resistance exercise demonstrated significant positive effects on the psychological dimensions of lung function and QOL in this population. Notably, the recovery of lung function and the improvement of QOL are both important factors for clinical outcomes in lung cancer patients [36]. It has been found that postoperative lung cancer patients may have damaged lung tissues and decreased lung function, which increases the risk of respiratory infections in patients [37]. Therefore, good lung function recovery is important for improving the QOL and prolonging the survival of lung cancer patients. Numerous studies have been conducted on the effects of exercise on postoperative recovery in NSCLC patients [4, 18, 32, 38], but the results vary widely. In this study, we summarized the recently published studies and found that long-term exercise can improve the postoperative recovery (lung function, exercise capacity, and QOL) of NSCLC patients and alleviate dyspnea to a certain extent.

We explored lung function, exercise capacity, QOL, and cancer-related symptoms. Because evidence suggests that FEV1, VO_2_peak, and QOL are directly or indirectly associated with survival in NSCLC patients [36, 39, 40]. Reduced FEV1 may diminish the lung's ability to remove air, leading to decreased gas exchange efficiency, predisposing to hypoxemia and hypercapnia, and increasing the risk of postoperative complications [37]. Reduced VO_2_peak decreases the ability of the cardiopulmonary system to deliver oxygen to the tissues, affecting the oxygen supply to tissues throughout the body [41]. Pneumonectomy may lead to poorer physical, social, and psychological well-being [9, 10]. Therefore, there is a need to improve the abnormally lowered FEV1, VO_2_peak, and QOL in postoperative NSCLC patients. Our study evinced that exercise significantly elevated FEV1, VO_2_peak, and QOL in postoperative NSCLC patients, thereby effectively improving postoperative recovery status and the utilization of oxygen by FEV1 and VO_2_peak. Improvement of postoperative recovery status can effectively prevent NSCLC patients from developing complications or prevent the aggravation of existing complications, and accelerate the recovery process to a certain extent.

According to our analysis, no overall improvement was revealed in lung function indices (FEV1, FEV) after exercise training. However, subgroup analyses by different types of exercise evinced noteworthy findings. Three studies combining aerobic and resistance exercise reported an improvement in FEV1, while another study also demonstrated an enhancement in FEV1 after combining respiratory and other exercises. Only one article was available in the HIIT subgroup, which disclosed significant results (p < 0.05); however, the value obtained was negative, suggesting a potential adverse effect of this form of exercise on lung function. Certain studies have reported the benefits of HIIT in improving FEV1 among lung cancer patients; even perioperative HIIT has shown favorable outcomes for FEV1 [42]. Given the limited number of included articles for FEV1, further research would be valuable to elucidate existing findings.

Following exercise interventions, our analyses demonstrated improvements in exercise capacity indices, including 6MWD and VO_2_peak. Specifically, the effect size of 6MWD (MD = 35.80 m) exceeded the minimum significant difference range for lung cancer patients (i.e., 22–42 m) [43], indicating clinical relevance. Subgroup analyses proved that combined aerobic and resistance exercises markedly improved 6MWD, but it was not significantly improved by HIIT and respiratory exercises combined with other exercises. There was moderate heterogeneity between the aerobic and resistance combined subgroups, which may be attributed to differences in intervention methods, duration, or preoperative health status of patients. Furthermore, there were insufficient data to support comparisons between respiratory exercises combined with other exercise subgroups and control groups; however, two articles [31, 32] reported favorable effects of breathing training combined with other exercises on exercise capacity, as evidenced by elevated levels of 6MWD compared to controls. Given these findings, further studies are recommended to fully substantiate the effectiveness of breathing combined with other exercises on exercise capacity index 6MWD among NSCLC patients.

The combined results elicited that exercise improved VO_2_peak. The VO_2_ peak effect size was found in this study (MD = 1.05). For every 1 mL/kg/min increase in VO_2_peak in NSCLC patients, there is a 4% reduction in all-cause mortality [44]. Another study reported [45] that HIIT increased VO_2_peak in preoperative (4–6 weeks of training) [46] and postoperative (14 weeks of training) [47] NSCLC patients by 2.4 mL/kg 1/min 1 (14.6%) and 1.7 mL/kg 1/min 1 (11%), respectively. A combination of aerobic and resistance training maximizes VO_2_peak levels in postoperative NSCLC patients [48]. Thus, exercise improves VO_2_peak in postoperative NSCLC patients. In addition, skeletal muscle function has important prognostic value in NSCLC [49]. Our analyses suggest that exercise (excluding respiratory exercise) can strengthen the quadriceps. However, the moderate heterogeneity in the quadriceps analysis may be due to different methods and durations of exercise, as well as the patient's preoperative health status. Postoperative NSCLC patients tend to be more likely to improve muscle function by increasing their VO_2_peak [50].

Two evaluation methods assessed QOL: the SF-36 and the EORTC QLQ-C30. According to the combined results, exercise training improved the SF-36 body domain scores in NSCLC patients after pneumonectomy. The study found a 3.10-point difference in HRQoL physical domain scores between groups within the median SF-36 interval (i.e., 3–5 points) [51]. However, mental domain scores for HRQoL were not improved. Subgroup analyses by different exercise types revealed new findings. Four studies combining aerobic and resistance exercises reported improvements in the mental domains. However, there was no significant improvement in the two studies in the HIIT subgroup. One of the studies [28] found no marked difference between the HIIT group and the control group in mental domain values. Another trial [26] analyzed the effects of HIIT and found considerable improvements in the psychological domains. Wang et al. concluded [52] that resistance training in the postoperative period in conjunction with HIIT is particularly effective for improving QOL. Due to the small number of psychological domain indicators, further research may be needed to address this issue.

By taking measurements with the EORTC QLQ-C30, we found that exercise could improve physical functions. However, no improvement was observed in overall health using the EORTC QLQ-C30., indicating that exercise training alone has a limited impact on this domain. Therefore, it is necessary to combine exercise training with other interventions by a multidisciplinary team, including various health professionals, to achieve more comprehensive improvements.

Dyspnea is a common symptom in lung cancer patients in the early and middle stages. After pneumonectomy, dyspnea, physical activity, exercise tolerance, climbing ability, and QOL may worsen and remain impaired for 6 months or more [9, 53–56]. In addition, adjuvant therapies (e.g., chemotherapy or radiation) may negatively affect patients' symptoms and physical status, similar to those of other cancer patients [54]. Bailey et al. [57] have reported that because medications do not completely relieve symptoms and increase the risk of side effects, future research must focus on promising non-pharmacologic interventions to manage dyspnea. Our study suggests that a combination of exercise and breathing training may be a promising non-pharmacological intervention. It can increase diaphragmatic activity, improve alveolar ventilation, reduce energy expenditure during respiration, and alleviate shortness of breath in lung cancer patients [58]. Therefore, this method may be an effective method to control dyspnea in NSCLC patients after pneumonectomy.

The combined results showed no change in fatigue levels after exercise training. Interventional studies have reported [24, 27–29] no change in fatigue levels after targeted exercise programs. A trial randomized 17 NSCLC stage I–III A patients 6–10 weeks after lobectomy into an exercise group (8 weeks of aerobic and resistance training) and a control group (usual care). After intervention, no significant changes in fatigue were found in either group [28]. In contrast, Quist reported [30] that early postoperative exercise reduced fatigue in patients with operable NSCLC. Although these findings seem to support the benefit of exercise on fatigue in postoperative NSCLC patients, based on our results, we are still unable to conclude that exercise significantly affects fatigue. Therefore, further studies are required to explore the true contribution of exercise to fatigue in postoperative NSCLC.

Among the 12 included studies, various interventions were employed, encompassing aerobic exercise, resistance exercise, and HIIT. While aerobic exercise and resistance training were more commonly utilized, additional interventions such as respiratory muscle training (RMT) and combinations of exercises were also implemented. Pneumonectomy can result in significant reductions in exercise capacity, respiratory muscle strength, lung volumes, and HRQoL among patients. However, previous research demonstrated that exercise training could enhance both exercise capacity and HRQoL in individuals with various chronic diseases, including COPD [59] and heart failure [60], as well as those diagnosed with prostate or breast cancer [61, 62]. Moreover, programs involving exercise training reported similar benefits for NSCLC patients after pneumonectomy [20]. The combination of aerobic and resistance training enhanced VO_2_peak, while the complementary physiological adaptations resulting from combined training promote cardiovascular oxygen delivery, skeletal muscle oxidative phosphorylation, muscle strength, and optimal fiber type composition. Consequently, this improved muscular endurance, reduced fatigue, elevated exercise metabolic waste thresholds, and decreased ventilatory requirements during physical activity [36]. On the other hand, HIIT induced a protective cardiorespiratory phenotype while improving oxygen extraction from skeletal muscle by increasing capillary density and mitochondrial oxidative capacity [63, 64]. Studies demonstrated that HIIT intervention in lung cancer patients enhanced their VO_2_peak, which in turn improved circulatory, respiratory, and muscular functions, consequently enhancing exercise capacity and lung function and relieving dyspnea [50]. Moreover, RMT, comprising inspiratory muscle training (IMT) and expiratory muscle training (EMT), either alone or combined with other exercises, represented an innovative and highly valuable physical activity therapy for patients undergoing major surgery. Zou et al. discovered that the combination of RMT and aerobic exercise was linked to decreased postoperative hospital stay and reduced incidence of postoperative complications while increasing exercise capacity along with FEV1 and FEV1/FVC values [32]. Therefore, the combination of RMT, especially IMT, and other exercises should be regarded as one of the optimal exercise rehabilitation treatments for patients undergoing pre- and postoperative pneumonectomy. It is crucial to investigate the effects of the combination of RMT and other exercises on the clinical outcomes of patients undergoing pneumonectomy.

### Limitations

Our meta-analysis has several limitations. First, many factors may contribute to clinical heterogeneity, such as differences in procedures, interventions, and control group characteristics. Second, due to differences in intervention methods, it is necessary to clarify which intervention specifically produced the overall effect. This issue requires further research. Third, due to insufficient supporting data, we did not perform subgroup analyses by VO_2_peak based on exercise training and the combination of exercise and breathing exercises. We did not analyze essential metrics such as inspiratory and expiratory muscle pressures. This issue also needs to be addressed in future studies.

## Conclusions and suggestions

This paper illustrates the benefits of exercise for NSCLC patients in improving exercise capacity, lung function, and QOL in selected domains, enhancing quadriceps function, and relieving dyspnea. However, we cannot draw rigorous conclusions about the effects of different exercise modalities on exercise capacity in NSCLC patients due to the lack of in-depth exploration. Given the paucity of data on outcomes other than exercise capacity (including QOL and various symptoms), our study focused on analyzing lung function, exercise capacity, QOL, quadriceps function, dyspnea, and fatigue. Future studies should focus on other factors influencing cardiovascular adaptive capacity and respiratory muscle function in NSCLC patients. The effects of combining exercise training with other interventions delivered by a multidisciplinary team (e.g., psychologists, etc.) also need to be integrated into future RCTs.

## Data Availability

All data supporting the findings of this study are available within the paper and its Supplementary materials.
